# Lessons learned from Taiwan’s response to the COVID-19 pandemic: successes, challenges, and implications for future pandemics

**DOI:** 10.1093/eurpub/ckae185

**Published:** 2024-11-20

**Authors:** Vivian Chia-Rong Hsieh, Min-Hua Tsai, Hua-Chueh Chiang, Ming-Yi Weng

**Affiliations:** Department of Health Services Administration, China Medical University, Taichung, Taiwan; Department of Health Services Administration, China Medical University, Taichung, Taiwan; Department of Health Services Administration, China Medical University, Taichung, Taiwan; Department of Health Services Administration, China Medical University, Taichung, Taiwan

## Abstract

This study aims to provide an investigation of the containment and mitigation strategies encompassing the entirety of the pandemic in Taiwan. This descriptive, observational study used COVID-19 data from Taiwan, Japan, and South Korea, and analysed news releases from the Taiwanese health authority. Statistics provided evidence of outbreak severity through infection and mortality rates, while qualitative results from the document review offered insights on the actions taken by the government chronologically from 2 February 2020 to 31 December 2022. All three countries experienced significant infection peaks in 2022. Taiwan had two distinct peaks, one in late May and another in October. South Korea had a single, high peak in late March, while Japan experienced multiple smaller waves, the biggest wave in August. Similarly, weekly mortality rates peaked in 2022 for all three countries after a surge in their infected cases, with Taiwan (5.15/100 000) and South Korea (4.69/100 000) having higher rates than Japan (1.65/100 000). Results from qualitative analysis showed that Taiwan’s early containment measures might have delayed the epidemic curve, allowing time for better preparation and proactive responses. However, the lack of a clear transition plan and the vulnerability of the elderly population contributed to higher mortality and infection rates. Despite ongoing challenges, Taiwan avoided nationwide lockdowns and relied on targeted restrictions to control transmission of the virus. Results of this article offer the narratives, reflections, and experiences from the case of Taiwan which may potentially present promising opportunities for impact in other settings and for future pandemics.

## Introduction

Presently, as we go about our daily lives and routine activities, it is difficult to imagine that we endured over two years of the COVID-19 pandemic. Being an island society geographically situated in close proximity to the first reported case in Wuhan, as well as its close economic ties with China, Taiwan faced an immediate risk to a spread of the outbreak. Thanks to the vigilant alertness of a health officer at the Centers for Disease Control (CDC), the news was quickly identified from social media monitoring, leading to immediate action for the implementation of relevant measures [1]. Drawing from the valuable lessons learned from the SARS outbreak in 2003 and the H1N1 flu pandemic in 2009, which laid a strong foundation for preparedness in the face of COVID-19 in 2020, Taiwanese authorities understood the importance of not only being hypersensitive to mysterious illnesses but also the importance of rapid control measures, effective crisis management, and most importantly, adopting a systematic approach to disease control and mitigating public anxiety [1–5]. In contrast, many other countries did not experience both of these previous public health crises.

Following the initial reported cases, everyday life and economic activities swiftly resumed in Taiwan and continued throughout the majority of 2020 and the first half of 2021, while many parts of the world experienced business and school lockdowns and disruptions to people’s daily routines [6–8]. However, by around May 2021, signs of public complacency and a return to pre-outbreak routines began to emerge. The surge in infected cases, however, did not happen until May of 2022 after preventive measures were gradually being lifted to bring social and economic activities back to normal.

Amidst the pandemic, numerous research studies have been published evaluating the effectiveness of the measures adopted in Taiwan. In a study published very early on in the pandemic, it was reported that Taiwan demonstrated swift and organised responsiveness. This included categorising SARS-CoV-2 as a prioritised infectious disease, granting the government authority and activating the Central Epidemic Control Center (CECC) as the hub for policy coordination and dissemination [2]. Measures such as delaying the commencement of the school semester after the winter break, and the meticulous tracking of individuals who had travelled to and from China were implemented. As the pandemic progressed, Chao *et al.* [9] linked a considerable portion of Taiwan's effective pandemic mitigation to its implementation of the universal masking policy (UMP). The UMP not only served as a non-pharmacological approach to combatting the virus, but it also became an effective medium for active public participation, and a generator for spillover effects such as public trust, altruism, and solidarity. Stringent border control measures were also put into effect, involving the screening and monitoring of travellers' body temperature and symptoms prior to boarding. Incoming travellers displaying relevant symptoms were subject to a 14-day quarantine, with isolated transportation from the airport [5]. Citizens were advised not to travel abroad unless due to extreme circumstances such as medical emergencies or deaths in the family. The government's proactive adoption of disruptive technology, efficient contact tracing, stringent border controls, and fair distribution of essential resources emerged as pivotal factors in preventing high infection and death rates as seen in many parts of the world [5, 10].

Nevertheless, these findings solely reflect actions and outcomes observed during the early phases of the pandemic (specifically, 2020–2021). A comprehensive investigation of mitigation strategies and outcomes encompassing the entirety of the pandemic has yet to be analysed, including the temporal trend of the epidemic along with the policies implemented and situations from nearby countries.

The aim of this article is two-fold. First, we will analyse Taiwan’s COVID-19 infection, mortality, and case fatality rates (CFRs), comparing with neighbouring countries Japan and South Korea, to identify trends and patterns during the period from 2020 to 2022. Second, we aim to conduct a qualitative analysis of Taiwan's experiences in health system governance, people-centeredness, equity, and innovative responses to COVID-19. We hope by examining and reviewing closely into Taiwan’s pandemic response in hindsight, valuable lessons can be learned in the event of the next global pandemic.

## Methods

### Study approach

This was an investigation uniquely based on the COVID-19 pandemic which offered a rare opportunity to examine the performance of Taiwanese health system in response to a public health crisis. A descriptive, observational approach was adopted using COVID-19 statistics and document review of news statements and releases from the Taiwanese public health authority. Statistics provided evidence on the severity of the outbreak in terms of infection, mortality, and case fatality, while qualitative results from the document review offered insights on the actions taken by the government. Ethics approval for this type of study was not required by the institutional review board of our institute.

### Quantitative data

We obtained population-level data on COVID-19 infections and related deaths from the Taiwan CDC on a daily basis. Data were extracted from the contents of news reports released by the CECC on its website (https://www.cdc.gov.tw/En). Taiwan's CDC adapted its COVID-19 case definition throughout the pandemic, transitioning from lab-based testing to include rapid diagnostic tests as infections surged in late May 2022 [11].

For our analysis of Japan and South Korea, we retrieved relevant COVID-19 statistics (number of infections and deaths) from the publicly accessible Complete Our World in Data dataset, which is ultimately sourced from the World Health Organization (WHO) (https://ourworldindata.org/covid-cases) [12]. A confirmed COVID-19 case, according to the WHO, is a person with laboratory confirmation of COVID-19 infection. The WHO gathered information regarding confirmed COVID-19 cases and deaths by means of official communications in accordance with the International Health Regulations, supplemented by monitoring the official websites and social media accounts of health ministries [13].

Data were collected for a 2-year period from 2 February 2020 to 31 December 2022 and estimated on a daily basis and later converted into weekly data (i.e. from the week of 2 February 2020 to the week of 25 December 2022). All data were collected from open data platforms and were count data reflecting date of reporting instead of date of symptom onset.

### Qualitative data

Primary source of qualitative data was online news reports from the official website of the CDC Taiwan. These reports were chosen due to their authority and reliability in providing updates on policy implementation and interventions related to the COVID-19 pandemic in Taiwan. One researcher was responsible for daily extraction of relevant information from the selected online news reports. This included details such as the date of announcement, target population for each policy or intervention, and the proposed plans of action outlined in the reports. The extracted data were categorised based on their relevance to three themes: (1) health system governance, (2) people-centeredness and equity, and (3) transformation and innovation. This step involved identifying key elements within each report that corresponded to these themes.

After the initial extraction and categorisation, another researcher verified the accuracy and completeness of the collected data. Both researchers then collaborated to organise the information in a chronological manner, ensuring coherence and consistency in the dataset. Both qualitative and quantitative data collection process spanned from July 2022 to July 2023.

### Data analysis

For data analyses, we initially compiled the count data on a weekly basis, which included the number of infections and deaths. Infection and mortality rates were expressed as number of infected cases and deaths per 100 000 population, respectively, whereas CFR was presented as a percentage of deaths among confirmed cases in a given period. Subsequently, we represented these rates graphically on a timeline using Microsoft Excel. This allowed us to visualise the pandemic over the specified time period. Secondly, qualitative data were systematically organised, with notable events plotted on a timeline to offer a qualitative overview. Furthermore, as described above, a content analysis was performed by two researchers who categorised action plans according to predefined themes and dates, evaluating their strengths and weaknesses. Our approach enabled a thorough understanding of the pandemic's progression, both quantitatively and qualitatively.

The study period between 2 February 2020 and 31 December 2022 was distinctively divided into three phases: early, vaccination, and easing phase. The ‘early’ phase started in the week of 2 February 2020, shortly after the WHO declared the novel coronavirus outbreak a public health emergency of international concern, and ended in the week of 4 January 2021. The ‘vaccination’ phase started in the week of 5 January 2021, following the emergency use validation of the Pfizer/BioNTech vaccine by the WHO, and ended on the week of 25 November 2021. Vaccination rollout began in Taiwan in March 2021 for healthcare workers, expanding gradually to other groups including government-employed epidemic prevention personnel and high-risk workers. The ‘easing’ phase commenced in the week of 26 November 2021, marked by the relaxation of prevention measures, and concluded in the week of 31 December 2022.

## Results

**Figure 1.**
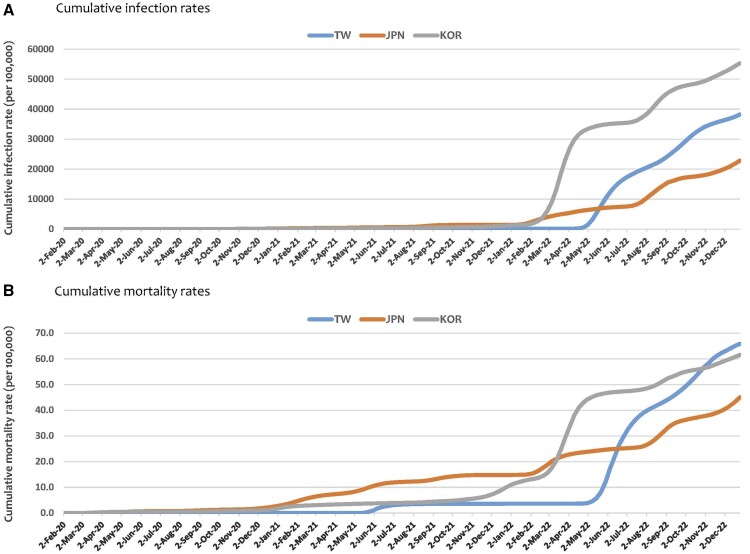
Cumulative infection and mortality rates (per 100 000 population) in Taiwan, Japan, and South Korea from the week of 2 February 2020 to the week of 25 December 2022 (TW—Taiwan; JPN—Japan; KOR—South Korea).

Figure 1 illustrates the cumulative infection and mortality rates in Taiwan, Japan, and South Korea throughout the study period. As of 31 December 2022, Taiwan had the second highest cumulative infection rate (38221.4/100 000), compared with South Korea (55309.5/100 000) and Japan (22804.4/100 000). However, Taiwan recorded the highest cumulative mortality rate (65.8/100 000) by the end of 2022, surpassing South Korea (61.5/100 000) and Japan (45.0/100 000). Notably, the infection and mortality curves exhibit a similar pattern, with a rise in mortality typically following a week after an increase in infections. Taiwan and South Korea experienced a sharp increase in cases and deaths in 2022 (starting around late April 2022 for Taiwan and February 2022 for South Korea). Conversely, Japan's cumulative infection and mortality rates rose more gradually throughout the study period.

**Figure 2.**
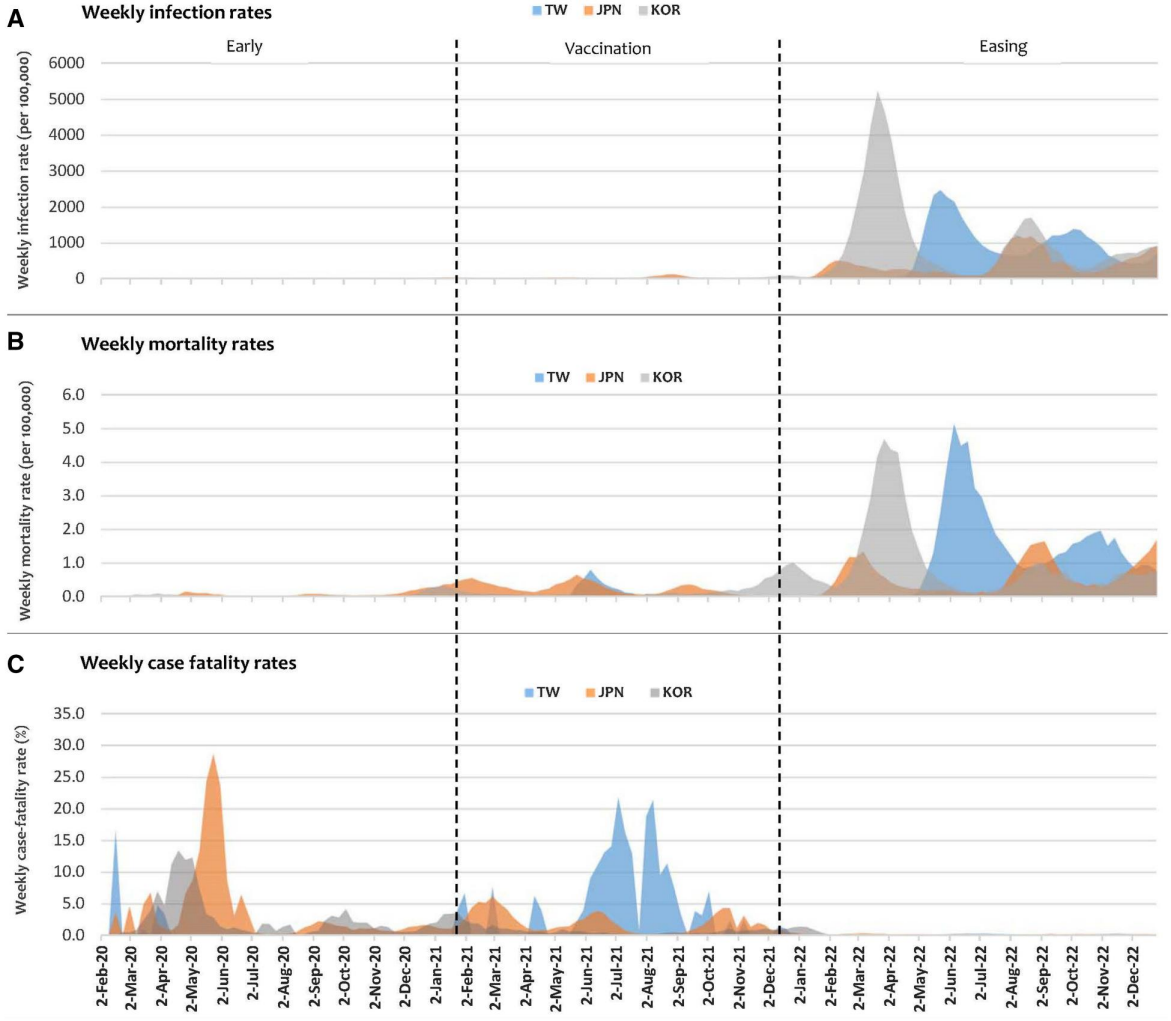
Weekly infection, mortality, and case fatality rates in Taiwan, Japan, and South Korea from the week of 2 February 2020 to the week of 25 December 2022 (TW—Taiwan; JPN—Japan; KOR—South Korea).

Weekly infection, mortality, and CFRs are depicted in Fig. 2. All three countries experienced significant peaks in 2022. Taiwan, in particular, had two distinct peaks of infection: one in late May 2022 (2481.8/100 000), coinciding with initiation of relaxed policies, and another in October 2022 (1393.7/100 000). Highest weekly infection rate in South Korea was seen in late March 2022 (5242.3/100 000). Japan, on the other hand, did not exhibit particularly high weekly infection rates, but rather multiple relatively smaller waves, the biggest wave in August 2022 (1207.7/100 000).

Similar with weekly mortality rates, most obvious waves for the three countries are observed in 2022. Highest in Taiwan was seen in early June 2022 (5.15/100 000), late March 2022 for South Korea (4.69/100 000), and early September 2022 for Japan (1.65/100 000).

Interestingly, trends in weekly CFRs for the countries are uniquely distinct from the above two metrics. Japan and South Korea experienced their highest CFRs in 2020 (28.7% in late May and 13.4% in late April, respectively), while Taiwan's peaks occurred in 2021 (21.8% and 21.4% between July and August) (Fig. 2). The overall CFRs by the end of 2022 were: Taiwan (0.17%), Japan (0.20%), and South Korea (0.11%) (data not shown). Timeline in the Supplementary Data details the major control strategies implemented by the Taiwanese authorities (Supplementary Fig. S1).

Results of the content analysis for qualitative data indicate the numerous important actions taken the Taiwanese health authority during the three phases (Table 1). In terms of health system governance, consistent measures were implemented across all three epidemic phases in Taiwan, including centralised policy management, border screening and control, closure and quarantine protocols, healthcare capacity adjustments, masking initiatives, and vaccination rollout. For example, within a month of the outbreak reported in Wuhan, China on 31 December 2019, Taiwan activated the CECC on 20 January 2020. It served as a centralised hub for pandemic information and policy coordination across government offices, operating under the rights of the Special Act for Prevention, Relief, and Revitalization Measures for Severe Pneumonia with Novel Pathogens. The first imported case was detected the following day. Subsequently, the CECC devised strategies related to healthcare capacity, public masking, border control, and social distancing, disseminating them through public announcements. On 6 February 2020, travellers from high-prevalence regions were postponed entry while travel advisory was given to the general public. In response to hospital infection incidents, the CECC provided protocol guidance and launched incentives program for healthcare professionals and providers for treating hospital-infected patients. Starting in March–April 2022, the government began easing control measures, considering Omicron's characteristics, international situations, and other factors, to resume normal socio-economic activities. Consequently, the criteria for releasing asymptomatic or mildly symptomatic COVID-19 cases from isolation and treatment began to lift in stages, moving towards a laissez-faire approach.

**Table 1.** Major plans of action and preparedness categorised chronologically into themes

	Governance		People-centeredness and equity		Transformation and innovation	
Early phase 1 February 2020 to 4 January 2021	2020-01-20	[Centralised policy] Central Epidemic Command Center (CECC) activated; all information on the pandemic and prevention measures was centralised and announced by the CECC	2020-04-01	[Social distancing] Social distancing measure phased-in guidelines established by the CECC to balance between public rights and domestic epidemic safety; to first encourage social courtesy and then to conduct mandatory measures	2020-01-27	[Contact tracing] National Health Insurance (NHI) smart card to alert travel history
	2020-02-06	[Border control] People residing in high-prevalence regions advised to postpone entry The general public is being urged to be vigilant in personal preventive measures when traveling in and out of the country. All cases confirmed by laboratory PCR test results	2020-04-30	[New lifestyle movement] Regular business and social activities can be performed as long as safety measures are adhered in outdoor activities and dining, maintaining social distances (1.5 m indoors, 1 m outdoors), personal hygiene, real-name registration, and crowd control, ensure a safe consumer experience	2020-02-06	[Face mask rationing 1.0] Surgical mask rationing for the public with NHI card in pharmacies and public health bureaus
	2020-02-20	[Closure guidelines] Criteria for school suspension established and announced	2020-11-25	[Financial risk protection] National health insurance covers COVID-19 medical expenses incurred abroad for its beneficiaries, up to a limit based on average costs at local hospitals and clinics	2020-03-12	[Face mask rationing 2.0] Online purchase of face masks available *via* NHI smart card or NHI mobile app
	2020-03-19	[Mandatory quarantine] All inbound passengers subject to 14-day quarantine upon arrival (maximum fine of 1 million NTD)			2020-05-28	[Contact tracing and privacy protection] For privacy protection and contact tracing purposes, venues collecting personal data must be solely for epidemic investigation purpose only, with designated handling staff and be kept for a maximum of 28 days
	2020-04-07	[Healthcare capacity] Six major strategies implemented to expand and reinforce healthcare capacity of hospitals				
	2020-12-01	[Public masking] People should wear masks in eight major types of venues/activities: healthcare, public transportation, daily consumption, education, exhibitions/sports events, leisure/entertainment, religious ceremonies, and government/public offices. Those who refuse to comply will be fined				
Vaccination phase 5 January 2021 to 25 November 2021	2021-01-22	[Healthcare capacity] In response to hospital infection incidents, the CECC initiated the incentives program for healthcare professionals and providers for treating hospital-infected patients, aka ‘Taoyuan Hospital Special Project’	2021-02-23	[Communication] The ‘1922 Epidemic Reporting and Consultation Service Center’ was established in 2004 in response to SARS in 2003 to provide 24-h toll-free epidemic reporting and infectious disease consultation services for everyone, including individuals living in the country, overseas, speaking English, and hearing-impaired individuals	2021-01-06	[Data protection] Operation ‘Electronic Fence 2.0’ initiated to help protect personal data while collecting epidemic prevention information
	2021-03-22	[Vaccination rollout] AstraZeneca COVID-19 vaccine rollout began for healthcare workers, expanding gradually to other categories including government-employed epidemic prevention personnel and high-risk workers	2021-03-22	[Inclusion and diversity] The ‘Taiwan V-Watch’ COVID-19 vaccine health reporting system was launched to track individuals' post-vaccination health. The system calculates the next vaccination date and sends advance reminders for better scheduling	2021-05-14	[Public alert] The ‘Taiwan Social Distancing App’ was launched to help users keep track of the spread of the epidemic by detecting the distance and time of indirect contact between users. It was designed to protect privacy and did not require users to register an account or provide any personal information
	2021-05-16	[Healthcare capacity] CECC announced four major healthcare contingency plans in response to the domestic community transmission, including delaying non-urgent medical treatments and procedures and screening patients for COVID-19 before they were admitted to hospitals and clinics	2021-05-05	[Inclusion and diversity] Unpaid COVID-19 vaccine leave implemented to encourage vaccination uptake for the working population	2021-07-26	[Epidemic investigation] The ‘Epidemic Investigation Assistance Platform’ was launched to facilitate epidemic investigation work with user-friendly interfaces and dynamic tools to track confirmed cases' activities
	2021-05-19	[Centralised policy] Nationwide epidemic alert raised to Level 3 with stricter prevention measures: no wedding banquets and public funeral ceremonies, take-out only policy for restaurants, enhanced crowd control in supermarkets and stores, and suspension of religious gatherings	2021-11-01	[Inclusion and diversity] Home quarantine measures revised ahead of the Lunar New Year in response to the increased influx of overseas citizens returning home to their families		
	2021-06-12	[Vaccination rollout] First batch of Moderna vaccine (150 000 doses) offered to all Category 1 individuals for vaccination, while maintaining strict border controls				
Easing phase 26 November 2021 to 31 December 2022	2022-03-01	[Centralised policy] Following an assessment of the epidemic status, including Omicron variant characteristics, vaccination rates, healthcare capacity, and international prevention measures, the government moderately relaxed prevention measures to uphold socio-economic activities and effective risk control	2022-05-28	[Inclusion and diversity] To protect residents in care facilities and vulnerable populations (including homeless and elderly living alone), complimentary home antigen rapid test kits were offered to both these facilities and individuals	2022-01-21	[Mobility and access] The ‘Digital COVID-19 Health Certificate’ became accessible for verifying vaccination status or negative test results, serving as a health certificate for entry into over 60 countries
	2022-04-03	[Centralised policy] Conditions for discharging asymptomatic or mildly symptomatic COVID-19 cases from isolation and treatment began to relax in phases (towards laissez-faire policy)			2022-03-08	[Mobility and access] To facilitate easy public access to COVID-19 vaccination sites, the CECC compiled weekly location data from local health bureaus and updated vaccine map on the CDC website
	2022-05-05	[Case definition] Individuals in home isolation/quarantine who tested positive on rapid antigen tests were defined as cases after inspection by healthcare professionals (i.e. no PCR test required)				
	2022-06-15	[Border control] Gradual border opening, shortened quarantine duration, and regulated inbound arrivals				
	2022-10-13	[Border control] Inbound travellers exempted from home quarantine and required to undergo ‘7-day self-health management’. COVID-19 travel advisory level lowered to Level 2				
	2022-11-28	[Public masking] Moderate relaxation of preventive measures such as mask-wearing				

For people-centeredness and equity, the early phase emphasised social distancing, adopting new lifestyle practices, and providing financial risk protection for the needed. Meanwhile, the vaccination and easing phases focused on effective communication, inclusion, and diversity. The new lifestyle movement encouraged the continuation of regular business and social activities, provided safety measures were followed. This included maintaining social distancing (1.5 m indoors, 1 m outdoors), adhering to personal hygiene practices including hand-washing and/or hand-sanitising, implementing real-name registration, and enforcing crowd control in public settings. Additionally, the National Health Insurance (NHI) reimbursed COVID-19 medical expenses incurred abroad, protecting patients from excessive healthcare spending due to necessary overseas travel. Measures for inclusion and diversity included allowing COVID-19 vaccine leave to promote vaccination uptake among the workforce, revising home quarantine protocols ahead of the Lunar New Year to accommodate the increased influx of overseas citizens, and providing complimentary home antigen rapid test kits to care facilities and vulnerable populations.

For transformation and innovation, initiatives focused on contact tracing, face mask rationing, data and privacy protection, data protection, and epidemic investigation were implemented during both the early and vaccination phases. This involved leveraging the NHI smart card for travel history alerts and face mask distribution at pharmacies and public health bureaus. Over time, this evolved into online face mask purchases via the NHI smart card or mobile app. In the easing phase, efforts were made to enhance mobility and accessibility to vaccination sites by compiling weekly location data from local health bureaus and updating the vaccine availability map on the CDC website. Moreover, measures were taken to ensure data protection, including the introduction of ‘Electronic Fence 2.0’ to safeguard personal information during epidemic prevention data collection.

Strengths and weaknesses of Taiwan’s prevention efforts are summarised in Table 2. Taiwan's COVID-19 prevention efforts have demonstrated strengths in early containment measures such as border controls, widespread mask usage, and efficient contact tracing. Furthermore, the use of technology such as mobile apps and NHI smart cards for digital contact tracing and health monitoring was effective. However, challenges during this period included limited vaccine availability in 2021, a lack of a clear post-restriction transition plan, and stigma-related barriers to accessing support services. Concerns were also raised regarding privacy and data security due to the use of digital tools for contact tracing.

**Table 2.** Strengths and weaknesses of Taiwan’s COVID-19 prevention efforts

	Strengths	Weaknesses
Governance	• Authorities demonstrated effectiveness in implementing timely and decisive measures, such as border controls, contact tracing, and isolation protocols, under the CECC • Centralised coordination and effective dissemination of information, including penalties for fake news or misinformation and distribution of face masks, facilitated a swift and unified response to the pandemic	• Despite the centralised approach, occasional communication gaps and inconsistencies in policy implementation were observed, leading to public confusion and compliance issues • There were also challenges in vaccine purchase and procurement that led to public concern and slowed the rollout process • The easing of restrictions in March and April 2022, amid the Omicron surge, potentially lacked a clear transition plan and support measures, leading to a healthcare system overwhelmed by a rapid increase in the number of cases
People-centeredness and equity	• Taiwan prioritised the health and safety of its citizens, evidenced by widespread access to testing, healthcare, quarantine facilities, and work absence support to both the patients and caregivers • The government's transparent communication and public education campaigns fostered a sense of shared responsibility among citizens, such as the ‘I Am Okay, You First’ campaign amid mask shortages to ensure mask access for those in need	• Border control measures restricted entry for many individuals from abroad, potentially limiting access to healthcare and support for certain populations • Stigma associated with occupation and ethnic background may have hindered equitable access to resources and support services for marginalised groups
Transformation and innovation	Technology was creatively leveraged to bolster the efficiency of surveillance and containment efforts, including the development of mobile apps for distributing face masks and scheduling vaccination appointments, as well as digital contact tracing apps and online platforms for health monitoring and information dissemination	• Concerns about data privacy and security arose due to the use of digital tools for routine contact tracing • Collective data including vaccination coverage, number of cases and number of deaths, were not provided on an open platform for the duration of the pandemic, impeding public awareness and research efforts

## Discussion

COVID-19 has yielded varying outcomes across different settings. Between 2020 and 2022, Taiwan experienced the highest cumulative mortality rate among three countries, but its cumulative infection rate was lower than South Korea's. Japan, on the other hand, had the lowest cumulative infection and mortality rates but the highest overall CFR.

Taiwan, with a population of 23.4 million and a density of 647 people per square kilometre, has an elderly (≥65 years) population exceeding 18% [14]. Its early and stringent border controls and contact tracing effectively kept the number of COVID-19 cases low from 2020 to 2021. An inevitable surge in infections, however, occurred following relaxation of preventive measures in April 2022. Unlike Japan and South Korea (with their infection and mortality peaks in February–April and August–September 2022), Taiwan experienced two distinct waves sometime later in 2022 as a result of widespread community transmission: Omicron BA.1/BA.2 in April–August and Omicron BA.5 in August–December. Mortality rates were highest during the first wave, particularly among the elderly [11].

One plausible explanation is that early containment measures delayed, rather than averted, the epidemic curve [15]. The extended period of low cases provided ample time for preparation and developing healthcare contingency plans, enabling authorities to proactively manage responses well in advance. At the same time, both the healthcare system and the public slowly adapted to the new social norms of hand sanitization, social distancing, and mask-wearing, further strengthening preventive effects. Despite initial success, as restrictions ended and a public desire to return to normal life might have triggered a rapid rebound in infections, especially among those who had not been infected before. This, coupled with the vulnerability of the elderly population and the lack of a clear transition plan, which led to a suddenly overwhelmed healthcare system, might have contributed to higher cumulative mortality and infection rates compared to other countries. High infection rates were similarly observed in South Korea during late March 2022 after easing restrictions amid the highly transmissible Omicron variant [16].

### Health system governance

For health system governance, the CECC relied on public health advice from a team of science and medical advisers, backed by the president, and supported by Acts such as the Communicable Disease Control Act and the Special Act for Prevention, Relief, and Revitalization Measures for Severe Pneumonia with Novel Pathogens [17]. Not only did it demonstrate effectiveness in implementing timely and decisive measures such as border control and quarantine protocols, it was also responsible for coordinating policy from the local to the national level [4]. Experience from the SARS pandemic in 2003 led to centralised coordination and effective information dissemination, including penalties for misinformation and face mask distribution. This facilitated a swift and unified response to the pandemic [1–5].

Collaboration between the government and private sector was also instrumental. Notably, this collaboration facilitated the increased production of face masks, a critical resource, especially during the early stages of the pandemic. In South Korea, private hospitals and clinics cooperated with the public health system to actively report suspicious cases, provide vaccinations and hospital beds, contributing to a low mortality rate [16].

Despite the centralised approach, occasional communication gaps and inconsistencies in policy implementation were observed, leading to public confusion and compliance issues. For example, in May 2021, the CECC prioritised vaccination for airline crew members, resulting in subsequent criticism over slow vaccination progress among pilots and subsequent cluster infections among them [18]. In June 2021, another cluster infection was identified at an agricultural products marketing corporation that had frequent interactions with local markets and vendors. The outbreak began with an unreported infected case, hindering the initiation of containment measures [19].

There were also challenges in vaccine purchase and procurement that slowed the rollout process. Most of the general public in Taiwan did not receive their first dose of the vaccination until after the summer of 2021, leading to its high fatality rate during the vaccination phase. Limited vaccine availability resulted in long waits for vaccination appointments, even following eligibility announcements. Conversely, South Korea saw its vaccination rate reached 50% in September [16], whereas Japan reached 82.1% first-dose coverage by mid-August of that year [20]. However, since vaccines were not available until 2021, Japan experienced a high CFR early on in 2020, which might be attributed to its older population. In 2020, Japan had a proportion of people aged 65 and over of 28.6%, compared to 16.0% in South Korea and 16.1% in Taiwan.

### People-centeredness and equity

Taiwan prioritised the health and safety of its citizens, evidenced by widespread access to testing, healthcare, quarantine facilities, and work absence support to both the patients and caregivers. Protecting vulnerable populations and ensuring people-centred policies are particularly crucial at times of public health emergencies. Financial support partially relieved the socio-economic burden of the pandemic enabling individuals to adhere to preventive measures such as expenses incurred in quarantine hotels early on in the pandemic. Other forms of relief included financial aids to businesses, healthcare providers, and schools, tax incentives, and salary supplements for unemployed individuals [21]. At the global level, mask donations were made to other countries facing shortages and Taiwan received vaccine assistance from friendly allies during critical times [9, 22].

However, stigma associated with occupation and ethnic background also might have hindered equitable access to resources and support services for marginalised groups. In Japan, some patients faced potential refusal of treatment because not all hospitals were designated for COVID-19 care [23].

### Transformation and innovation

Technology was creatively leveraged to bolster the efficiency of surveillance and containment efforts, including the development of mobile apps for distributing face masks and scheduling vaccination appointments, as well as digital contact tracing apps and online platforms for health monitoring and information dissemination [4, 9]. Unsurprisingly, just like other countries, there were concerns about data privacy and security arose due to the use of digital tools for routine contact tracing [6].

It is important to note that the number of confirmed cases in this study may be lower than the actual number of infections due to limited testing and reporting biases. Hence, we relied on data from reputable and official sources such as Taiwan CDC and the WHO to address these limitations.

Overall, Taiwan's response was robust but faced ongoing challenges in balancing between public health measures and socio-economic activities, and adapting to evolving circumstances. In spite of these challenges, Taiwan did not impose any lockdown throughout the pandemic. Whilst rises in infection and deaths seemed inevitable, unique situations were observed in the three different settings examined. Each country's unique interventions and timing played a significant role in their epidemic development. Common considerations included community participation, partnerships, healthcare capacity, vaccine availability, demographics, and phase transition readiness.

## Supplementary Material

ckae185_Supplementary_Data

## Data Availability

The data underlying this article are available in the article.
