# Supplementary material for: Lessons learned from Taiwan’s response to the COVID-19 pandemic: successes, challenges, and implications for future pandemics
Source: Eur J Public Health. 2024 Nov 20;35(1):153–62. doi: 10.1093/eurpub/ckae185 (PMC11832153; doi:10.1093/eurpub/ckae185)
Supplement: ckae185_Supplementary_Data [file ckae185_supplementary_data.docx]

**Supplementary Data**

**Figure S1. Timeline of major control measures implemented by the Taiwanese authority from the week of February 2, 2020 to week of December 25, 2022.**

**Easing**

**Early**

**2022-03-01**

Preventive measures began to relax

**2021-05-16**

Contingency plans for hospital and clinics

**2021-05-19**

Nationwide level 3 epidemic alert with strict prevention measures

**2020-03-19**

Mandatory quarantine for all inbound travelers

**2020-02-06**

-Border control & screening

-Face mask rationing

**2021-01-22**

Incentives for healthcare providers

**2021-03-22**

Vaccine rollout began

**2020-05-28**

Privacy protection guidelines for contact tracing

**2020-04-30**

New lifestyle practices

**Vaccination**

Note: In the early phase, comprehensive and timely policies encompassed strengthened border control, quarantine and isolation protocols, school closures, mandatory mask-wearing, and healthcare capacity reinforcement. The following vaccination phase was marked by distribution of vaccines and the activation of healthcare contingency plans in response to local community spread. Finally, the easing period was characterized by the relaxation of preventive measures such as quarantine and border control policies, resulting in a significant surge in infection and mortality rates.
